# Postmortem neuropathology in COVID‐19: An update

**DOI:** 10.1111/bpa.13204

**Published:** 2023-08-11

**Authors:** David S. Younger

**Affiliations:** ^1^ Departments of Clinical Medicine and Neuroscience City University of New York Medical School New York New York USA

Dear Editor,

Between 2020 and 2021, Younger [1, 2] described the neuropathology of 144 decedents due to severe acute respiratory syndrome‐coronavirus‐2 (SARS‐CoV‐2) at the height of the 2019 coronavirus‐2 (COVID‐19) pandemic. The present study concerns an updated later cohort adding 150 new cases [3, 4, 5, 6, 7, 8, 9, 10, 11, 12, 13, 14, 15, 16, 17, 18, 19, 20]. There was a modest demographic shift in the updated later cohort. The study population comprised 12 children and 138 adults in a 2.6:1 ratio of males to females, of age range (7 months to 97 years) with 72% ≥50 years, compared to the earlier cohort in whom all were adults of older ages (>age 65 years in 79%). Serum cytokine and procoagulant levels were elevated in the vast majority of patients in life in both cohorts indicative of a cytokine storm, who were managed in an intensive care unit in 86% of cases (equally compared to the earlier cohort of 88%) until the time of death that occurred in a ratio of ≤10 days or more (0.78:1). There were seven children with COVID‐19 associated multisystem inflammatory syndrome (MIS‐C) (six patients) in the later cohort including one with angiographically negative small vessel childhood primary angiitis of the central nervous system (AN‐SV‐cPACNS) [15]. There was also a modest increase in several neuropathological aspects in the updated later cohort compared to the initial early cohort that included: Interstitial brainstem inflammation and neuronal loss (25% vs. 8%), focal or diffuse perivascular parenchymal T‐cells (17% vs. 7%), acute hemorrhagic or ischemic infarcts (25% and 11% vs. 2% and 7%), and hypoxic–ischemic changes and neuronal loss (25% vs. 19%). Positive detection of SARS‐CoV‐2 ribonucleic acid (RNA) by quantitative real‐time polymerase chain reaction (qRT‐PCR) decreased, while negative findings increased respectively (17% and 44% vs. 9% and 22%). Investigations applying commercially available antisense nucleocapsid and spike gene probes to formalin‐fixed paraffin‐embedded tissue with amplification of in situ hybridization (ISH) signals employing RNAscope™ [21], counterstained by hematoxylin and eosin with comparison to control antisense probes, failed to show positivity in parenchymal brainstem tissues but did show positivity in the adventitia of a meningeal vessel outside of the medulla [20]. Brain microglia activation so noted in 21% of cases, although rarely mentioned in the earlier cohort.

Several salient findings characterize the morbid features of COVID‐19 neurological illness. Elevated serum cytokines and procoagulant levels due to a cytokine storm likely contribute to critical illness, acute hemorrhagic, and thrombotic infarcts. Hypoxic–ischemic changes seen in up to a quarter of cases may not account for the myriad of neuropathological changes particularly indolent interstitial parenchymal cerebral or brainstem inflammation mediated predominantly by infiltrating CD8 T‐cells. Despite the overall generally favorable prognosis, systemic and CNS inflammation contributes to mortality of pediatric COVID‐19 neurological illness. Viral studies typically show negative results employing RNA isolation by qRT‐PCR, IHC, and ISH. Looking forward, the importance of performing autopsy studies resides in elucidating potentially early humoral and adaptive immune responses underlying the mechanisms of morbid neurological illness in COVID‐19.

Correlative brain imaging utilizing three‐dimensional surface projections of ^18^fluorodeoxyglucose positron emission tomography normalized to the whole brain and fused to non‐contrast magnetic resonance imaging with volumetric analysis not previously available at the onset of the COVID‐19 pandemic may reveal insights into the premorbid neuropathology of affected patients. Severely ill children with post‐acute SARS‐CoV‐2 (PASC) so studied show prominent cortical hypometabolism in widespread areas with volume loss related to the dysregulated post‐infectious immune response [22]. These neuroimaging findings in clinically correlative areas notably in the medial temporal lobe and hippocampi attesting to the duration and severity of neurological deficits in mood and neurocognition, appear to be influenced by the response of brain microglial that transition from surveilling mode to a reactive state changes in initiating and expanding neuroinflammation [23].

## CONFLICT OF INTEREST STATEMENT

The author has no conflicts of interest to report.

## Data Availability

Data sharing is not applicable to this article as no new data were created or analyzed in this study.
